# Preparation and Identification of Corn-Derived Bioactive Peptides with Triple Efficacy of ADH-Activating, XOD-Inhibiting and Antioxidant Activity

**DOI:** 10.3390/foods15061093

**Published:** 2026-03-20

**Authors:** Zifan Yuan, Wenfei Zhang, Jiajie Chang, Yunlong Chen, Yinglian Zhu, Qi Wang, Qingli Yang

**Affiliations:** College of Food Science and Engineering, Qingdao Agricultural University, No. 700 Changcheng Road, Qingdao 266109, China; yzfjzh@163.com (Z.Y.); zwfguiling@163.com (W.Z.); changjiajie2026@163.com (J.C.); yearschenyi@163.com (Y.C.); cjs52002@163.com (Y.Z.)

**Keywords:** corn peptide, ADH activation, XOD inhibition, antioxidation, computer-aided screening, molecular docking

## Abstract

The health risks associated with excessive alcohol consumption have emerged as a public health challenge, with alcohol-associated liver disease (ALD) and hyperuricemia (HUA) being particularly prominent health issues. Current treatments often have side effects, driving the need for safe, multi-target natural alternatives. Based on the dual barrier strategy of “metabolic regulation–antioxidant defense”, this study developed bioactive peptides from corn germ meal via enzymatic hydrolysis, which simultaneously activated alcohol dehydrogenase (ADH), inhibited xanthine oxidase (XOD), and exhibited antioxidative properties. The fraction <3 kDa emerged with stronger triple bioactivity while also demonstrating sensitivity to strong acids and enhanced activity under trypsin treatment in in vitro stability tests. A total of 841 unique peptides were obtained from purified peptide fractions. After computer-aided screening and molecular docking, three corn-derived peptides (LMFP, FEGLFR, and QLPSYR) were identified, which acted synergistically. Docking simulations revealed that they bind to ADH and XOD via hydrogen bonds and hydrophobic interactions, suggesting potential interactions with these enzymes that may influence their activity. The corn-derived bioactive peptides developed in this study may serve as potential resources for alleviating alcohol metabolism and hyperuricemia symptoms.

## 1. Introduction

ALD and HUA are two globally prevalent metabolic disorders. Excessive alcohol intake is a common risk factor for both diseases [1,2]. According to statistics, approximately 25% of adults worldwide suffer from hyperuricemia, and the prevalence of alcoholic liver disease is significantly higher among long-term drinkers [3]. Chronic heavy drinking induces hepatic fat accumulation, inflammation, and fibrosis [4]. Additionally, excessive alcohol consumption is associated with high purine intake, and lactate produced from ethanol metabolism competitively inhibits the excretion of uric acid (UA), leading to elevated serum UA levels [5,6]. The concurrent presence of ALD and HUA can create a vicious cycle in liver and kidney metabolism, and both share the core pathological pathway of oxidative damage–inflammation [7,8,9,10]. The large amount of reactive oxygen species (ROS) generated during alcohol metabolism serves not only as a direct driver of liver damage but also as an activator that promotes UA production [11]. Moreover, UA crystals and elevated UA levels can further trigger inflammatory signaling. HUA exacerbates inflammatory liver injury, whereas ALD reduces the capacity for UA excretion [12]. This interaction ultimately leads to irreversible organ damage. Therefore, strategies that simultaneously accelerate alcohol metabolism (via ADH activation), reduce UA production (via XOD inhibition), and alleviate oxidative stress (via antioxidant activity) may offer synergistic benefits by targeting interconnected pathological pathways. Currently, clinical medications for ALD mainly include alcohol metabolism-promoting drugs (e.g., metadoxine), antioxidant drugs (e.g., glutathione and silymarin), and hepatoprotective and anti-inflammatory drugs (e.g., polyene phosphatidylcholine and glycyrrhizinate preparations). For HUA, common drugs are mainly XOD inhibitors, including allopurinol and febuxostat. Overall, while current treatment options for ALD and HUA have diversified, these drugs generally act on single targets and are often associated with varying degrees of side effects. Therefore, the development of safe, effective, and multi-target natural therapeutics is an urgent need and an important direction in current research.

Bioactive peptides are food-derived protein peptides composed of 2–20 amino acids that are obtained through protease hydrolysis or microbial fermentation. They are characterized by their absence of side effects, safety, ease of absorption, and high biological activity. Various protein sources, including plant, dairy, marine, microalgal, and insect proteins, have been reported for the development of bioactive peptides [13,14]. The biological activities of these peptides are influenced by their amino acid features, such as charge, hydrophilicity, hydrophobicity, steric hindrance, and sequence order [15]. They exhibit diverse functional properties, including free radical scavenging [16], anti-inflammatory [17] and antimicrobial effects [18], blood glucose regulation [19], uric acid reduction [20], blood pressure modulation [21], and flavor enhancement [22]. Owing to their favorable functional profiles, bioactive peptides have become a research hotspot in the fields of biomedicine and food for special medical purposes.

This study utilized corn germ meal as the raw material to prepare corn peptides with the triple functions of “scavenging free radicals, activating ADH, and inhibiting XOD activity” through protease screening, hydrolysis process optimization, separation, purification, identification, and virtual screening. Finally, the docking sites and interaction forces between the bioactive peptides and target enzymes were determined using molecular docking tools. The perspective of this study differs from the traditional single-target approach, innovatively proposing a comprehensive “metabolism-promoting and oxidative defense” strategy. This study provides a basis for the preparation and screening of corn-derived peptides with triple efficacy, although further in vivo studies are needed to evaluate their physiological relevance.

## 2. Materials and Methods

### 2.1. Materials and Reagents

Corn germ meal (20 g protein/100 g) was purchased from a local market (Qingdao, China). Neutral protease, alkaline protease, papain, trypsin, 2,2-Diphenyl-1-picrylhydrazyl (DPPH), ADH (300 U/mg) and XOD (50 U/mg) were procured from Shanghai Yuanye Biotechnology Co., Ltd. (Shanghai, China). β-nicotinamide adenine dinucleotide (NAD^+^) and xanthine were obtained from Shanghai Macklin Biochemical Technology Co., Ltd. (Shanghai, China). Ultrafiltration centrifuge tubes were sourced from Merck Millipore Co., Ltd. (Burlington, MA, USA). Three peptides (LMFP, FEGLFR, and QLPSYR) (purity > 95%) were chemically synthesized by Sangon Biotechnology Co., Ltd. (Shanghai, China).

### 2.2. Enzymatic Hydrolysis of Corn-Derived Peptides

The 10% (*w*/*v*) corn germ meal solution was boiled for 10 min. After the corn solution was cooled, 1% (*w*/*w*) protease was added, followed by a 2 h hydrolysis under the optimal temperature and pH conditions for the protease. During this process, 0.1 M NaOH was added while stirring to maintain the system pH at the protease’s optimum. By heating the hydrolysate at 90 °C for 15 min, the enzymatic reaction was stopped. The enzymatic hydrolysate was centrifuged at 4000 r/min for 20 min, and the supernatant was retained.

### 2.3. Protease Screening

Corn germ meal was hydrolyzed using neutral protease, alkaline protease, papain, and trypsin. The degree of hydrolysis (DH), DPPH free radical scavenging rate, ADH activation rate, and XOD inhibition rate were used as evaluation indicators to screen for the most suitable protease.

### 2.4. Calculation of DH

The hydrolytic activity of protease is determined by the pH-Stat method [23,24], with the formula given below:

DH (%) = (C × V)/(a × m × h_tot_) × 100%

a = 10^(pH − pKa)/[1 + 10^(pH − pKa)]

Here, C is the concentration of the NaOH solution (mol/L); V is the volume of the NaOH solution (mL); a is the degree of dissociation of the α-amino groups; pH is the hydrolysis system pH; and pKa is the dissociation constant of the amino groups, where pKa = 7.8 + (298 − T)/(298 + T) × 2400. T is the temperature in Kelvins; m is the total mass of the protein substrate (g); h_tot_ is the total number of peptide bonds in the substrate protein (mmol/g); and for the corn protein, h_tot_ = 7.35.

### 2.5. ADH Activation Rate Assay

We employed an optimized protocol based on the method described by Xiao [25] to determine ADH activity. A blend of 50 μL peptide solution and 150 μL detection reagent (containing 22.4 mM sodium pyrophosphate buffer, 3.3% ethanol, and 7.8 mM NAD^+^) was equilibrated at 37 °C for 5 min, after which the assay was started by the addition of 50 μL ADH (0.2 U/mL). Absorbance at 340 nm was recorded every 10 s for 10 min, using distilled water in place of the sample as the negative control. To calculate the ADH activation rate, the initial reaction rates for the sample (Vs) and the negative control (Vo) were obtained as the first derivative at 0 min of their respective fitted kinetic curves. The ADH activation rate was then determined according to the following equation:

ADH activation rate (%) = (Vs − Vo)/Vo × 100%

### 2.6. XOD Inhibition Rate Assay

The testing method for the XOD inhibition rate was modified from a previous report [26]. A blend of 50 μL peptides and 50 μL of 0.02 U/mL xanthine oxidase was equilibrated at 25 °C for 5 min. Then, 150 μL of 0.48 mM xanthine was added, and the mixture was incubated at 25 °C for 25 min. Subsequently, 80 μL of 1 M HCl was added to terminate the reaction. At 290 nm, the absorbance of each group’s supernatant was recorded. The XOD inhibition rate was then calculated as follows:

XOD inhibition rate (%) = 1 − [(A − B)/(C − D)] × 100%

Here, A is the absorbance of the sample group (containing both peptide and enzyme); B is the absorbance of the no-enzyme control (peptide without XOD); C is the absorbance of the no-sample control (XOD without peptide); D is the absorbance of the blank control (neither peptide nor XOD).

### 2.7. Determination of DPPH Radical Scavenging Ability

A total of 500 μL of 1 mg/mL corn enzymatic hydrolysate was mixed with an equal volume (500 μL) of 0.1 mmol/L DPPH ethanol solution. The mixture was kept in the dark at 25 °C for 30 min, after which the absorbance at 517 nm was recorded as A_sample_. A blank was prepared by replacing the DPPH ethanol solution with an equal volume of pure ethanol, and its absorbance was recorded as A_blank_. A control was prepared by replacing the enzymatic hydrolysate with an equal volume of distilled water, and its absorbance was recorded as A_control_. The DPPH radical scavenging rate was then calculated as follows:

DPPH scavenging rate (%) = [(A_control_ − A_sample_ + A_blank_)/A_control_] × 100%

### 2.8. Hydrolysis Process Optimization

After selecting the optimal protease, the effects of different enzyme dosages (0.5%, 1%, 1.5%, 2%, 2.5%), hydrolysis times (1 h, 2 h, 3 h, 4 h, 5 h), and solid–liquid ratios (10%, 20%, 30%, 40%, 50%) on the DH, DPPH radical scavenging rate, ADH activation rate, and XOD inhibition rate were investigated to determine the optimal range for the enzymatic hydrolysis process.

Within the optimal range for enzymatic hydrolysis, with enzyme dosage (A), hydrolysis time (B), and the solid–liquid ratio (C) as independent variables, and using the DH (Y1), DPPH radical scavenging rate (Y2), ADH activation rate (Y3), and XOD inhibition rate (Y4) as response values, a response surface methodology experiment was designed employing the Box–Behnken Design in Design-Expert 13 software to determine the optimal process conditions for corn protein hydrolysis.

### 2.9. Separation and Purification of Corn Protease Hydrolysate

The corn protein enzymatic hydrolysate was filtered using 10 kDa and 3 kDa ultrafiltration tubes to obtain three fractions with molecular weights of >10 kDa, 3–10 kDa, and <3 kDa. The <3 kDa fraction was further purified using an ÄKTA pure protein purification system equipped with a Sephadex G-15 gel filtration column, and peptide fractions corresponding to different peak values were collected. Finally, the peptide fraction with the highest bioactivity was subjected to amino acid sequence identification by UPLC-MS/MS.

### 2.10. Stability Test of Corn Peptides (<3 kDa Fraction)

#### 2.10.1. Stability of Corn Peptides Under Different pH Conditions

A total of 5 mL of 1 mg/mL peptide solution (<3 kDa fraction) was adjusted to pHs 1, 3, 5, 7, 9, and 11 using 1 mol/L HCl or NaOH solution. The solutions were incubated at room temperature for 30 min, after which the pH of each sample was readjusted to pH 7. A control group consisted of the sample without pH adjustment. Deionized water was then added to bring the final volume of each solution to 20 mL. The DPPH scavenging rate, ADH activation rate, and XOD inhibition rate of each sample were subsequently measured.

#### 2.10.2. In Vitro Simulated Digestion

The preparation formula for simulated gastric juice and intestinal fluid was adapted from Berardi [27]. The preparation of simulated gastric fluid was as follows: 0.6 g of pepsin and 0.4 g of NaCl were dissolved in 200 mL water, and the pH of the solution was adjusted to pH2. The preparation of simulated intestinal fluid was as follows: 0.6 g of trypsin and 1.36 g of KH_2_PO_4_ were dissolved in 200 mL water, and the pH of the solution was adjusted to pH7.5.

A total of 10 mL of 1 mg/mL peptide solution (<3 kDa fraction) was pre-incubated with shaking in a 37 °C water bath for 5 min. Then, 5 mL of simulated gastric fluid, simulated intestinal fluid, or a combination of both (simulated gastric + intestinal fluid) was added separately. The mixtures were digested in the dark with shaking at 37 °C for 2 h. Enzymatic activity was stopped by heating in a 70 °C water bath for 10 min, and the pH of the samples was adjusted to pH7.0. The resulting mixtures were centrifuged (8000 r/min, 10 min). The supernatants obtained were labeled as the simulated gastric digestion (SGD), simulated intestinal digestion (SID), and simulated gastrointestinal digestion (SGID). A blank control was prepared without adding any simulated digestion fluid.

### 2.11. Computer-Aided Screening

From the mass spectrometry results, peptides were sequentially screened based on the following criteria [28,29,30]: molecular weight < 1000 Da, Average Local Confidence (ALC) > 80%, and relative peak area >10^5^. Subsequently, the virtual screening of the peptide sequences obtained from mass spectrometry was performed using online tools such as PeptideRanker (http://distilldeep.ucd.ie/PeptideRanker/, accessed on 10 March 2026), ToxinPred3.0 (https://webs.iiitd.edu.in/raghava/toxinpred3/prediction.php, accessed on 10 March 2026), and Peptide property calculator (http://www.innovagen.com/proteomics-tools, accessed on 10 March 2026).

### 2.12. Molecular Docking

The three-dimensional structure of DPPH (PubChem CID: 2735032) was obtained from the PubChem database. The 3D crystal structures of ADH (PDB ID: 5ENV) and XOD (PDB ID: 1N5X) were downloaded from the Protein Data Bank (PDB) database. The 3D structures of the corn peptides were drawn using ChemDraw 20.0 software. Molecular docking was performed using AutoDock Tools-1.5.6 software to investigate the binding energy, hydrogen bonds, and intermolecular interactions between the corn peptides and ADH or XOD. The molecular docking results were visualized using PyMOL2 software.

### 2.13. Statistical Analysis

All experiments were conducted at least three times. The experimental data was expressed as the mean ± standard deviation and analyzed by an ANOVA test using SPSS 19.0 software. *p* < 0.05 was significant among the groups.

## 3. Results

### 3.1. Screening of Proteases

**Figure 1:**
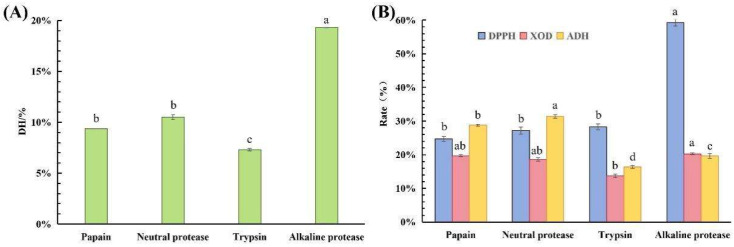
(**A**) Degree of hydrolysis of different proteases. (**B**) Biological activity of corn hydrolysates prepared by different proteases. Note: Different letters (a–d) indicate significant differences between groups (*p* < 0.05).

Different proteases possess distinct cleavage sites, amino acid preferences, and hydrolytic capacities [31]. Under identical experimental conditions, four proteases—papain, neutral protease, trypsin, and alkaline protease—were compared based on four indicators: the DH, ADH activation rate, XOD inhibition rate, and antioxidant activity. The DH results indicated that alkaline protease exhibited the strongest hydrolytic ability (Figure 1A). In terms of bioactivity, the hydrolysate of alkaline protease showed superior performance (Figure 1B). Therefore, alkaline protease was selected as the optimal protease for hydrolysis.

### 3.2. Enzymatic Hydrolysis Process Optimization

#### 3.2.1. Single-Factor Optimization Results

**Figure 2:**
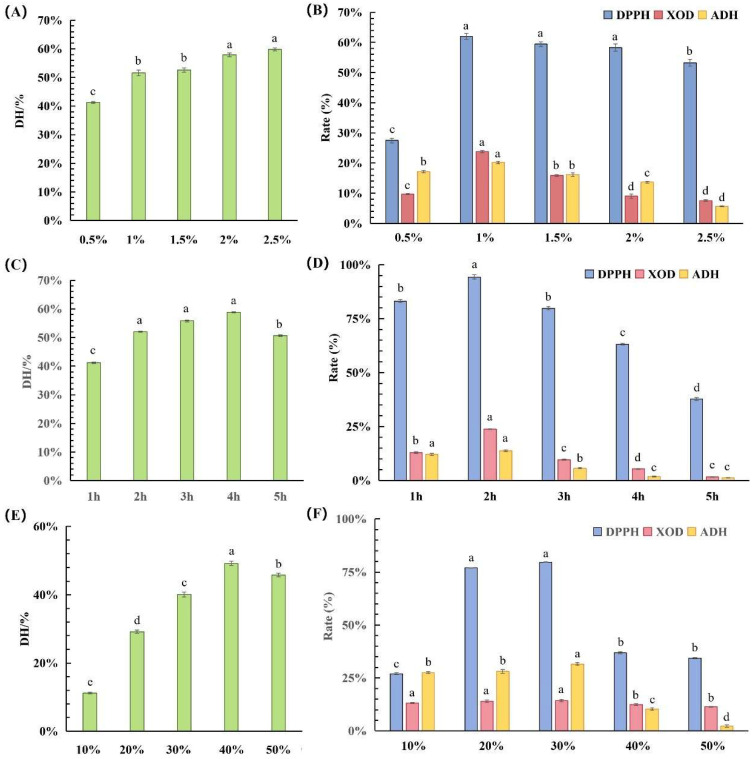
(**A**) DH of different enzyme dosages. (**B**) Bioactivity of corn hydrolysates prepared using different enzyme dosages. (**C**) DH of different hydrolysis times. (**D**) Bioactivity of corn hydrolysates prepared using different hydrolysis times. (**E**) DH of different solid–liquid ratios. (**F**) Bioactivity of corn hydrolysates prepared using different solid–liquid ratios. Note: Different letters (a–e) indicate significant differences between groups (*p* < 0.05).

The impacts of alkaline protease hydrolysis on corn germ meal were investigated using different enzyme dosages, hydrolysis times, and solid–liquid ratios. The optimal hydrolysis process range for alkaline protease was determined by evaluating four indicators: the DH, DPPH radical scavenging rate, ADH activation rate, and XOD inhibition rate. As shown in Figure 2, with an increasing enzyme dosage, hydrolysis time, and solid–liquid ratio, the DH exhibited an initial increase followed by a decrease, whereas the DPPH radical scavenging rate, ADH activation rate, and XOD inhibition rate exhibited a bell-shaped pattern. As the active sites of the protease gradually became occupied and protein hydrolysis proceeded, the product yield increased. When the remaining protein approached saturation, the rate of the DH gradually slowed down. The changes in the bioactivity of the hydrolysates were closely related to the molecular weight and exposure of the active groups. Appropriate hydrolysis can expose bioactive peptides, while excessive hydrolysis may further break these peptides into shorter fragments, disrupting key functional groups and thus reducing bioactivity. Thus, almost all indicators exhibited a bell-shaped pattern. The optimal ranges were a hydrolysis time of 1.5–3 h, enzyme dosage of 1–1.5%, and solid–liquid ratio of 20–35%.

#### 3.2.2. Response Surface Optimization Results

Using enzyme dosage (A), hydrolysis time (B), and the solid–liquid ratio (C) as independent variables, and the DH (Y1), DPPH radical scavenging rate (Y2), ADH activation rate (Y3), and XOD inhibition rate (Y4) as response values, the response surface results are as shown in Table 1. Regression analysis was performed using Design Expert software, and the fitted equations were as follows (Table 2, Table 3, Table 4 and Table 5):

Y1 = 46.21 + 2.12 A + 7.30 B + 6.26 C + 1.44 AB − 0.45 AC − 1.75 BC + 3.28 A^2^ − 7.22 B^2^ − 0.47 C^2^

Y2 = 61.33 − 3.72 A − 1.52 B + 0.9388 C + 1.64 AB − 2.93 AC + 2.36 BC − 25.36 A^2^ − 18.73 B^2^ − 17.69 C^2^

Y3 = 13.29 + 0.5350 A − 0.2463 B + 0.8487 C + 2.75 AB − 2.91 AC + 3.02 BC − 3.39 A^2^ − 4.58 B^2^ − 2.03 C^2^

Y4 = 48.90 + 1.37 A − 0.2825 B + 1.87 C − 0.8850 AB − 2.59 AC − 2.38 BC − 17.47 A^2^ − 16.73 B^2^ − 16.02 C^2^

The ANOVA tables showed that all model *p*-values were <0.01, indicating high significance, while the lack-of-fit *p*-values were >0.05, suggesting non-significance. This demonstrates that the regression equations fit the experimental results well. With R^2^ > 0.9, the models exhibited strong explanatory power; thus, the regression equations can be used to analyze and predict the actual experimental values. Although relatively higher coefficients of variation were observed for ADH activation (22.06%) and XOD inhibition (27.04%), reflecting the inherent sensitivity of enzyme-based bioassays, the models remained highly significant (*p* < 0.01) with a good fit, supporting the validity of the optimization results.

**Figure 3:**
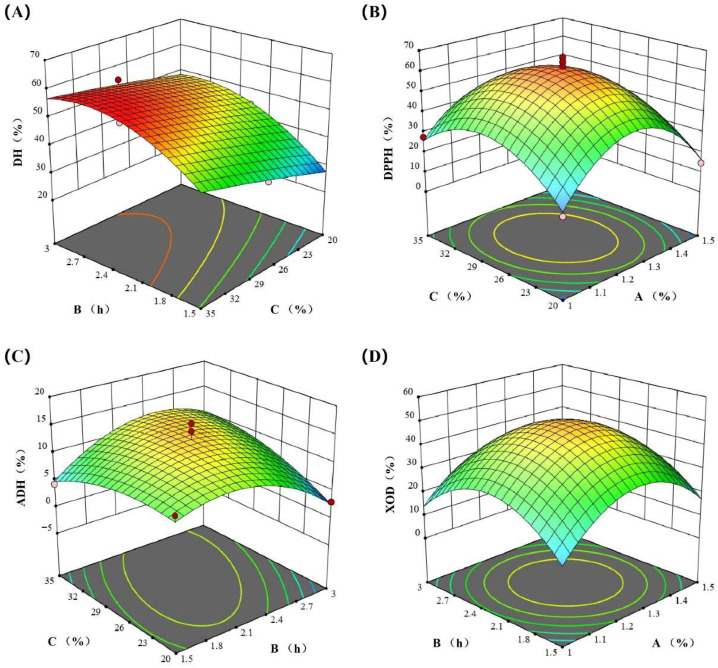
The response surface diagram of experimental factors: the (**A**) DH, (**B**) DPPH radical scavenging rate, (**C**) ADH activation rate, and (**D**) XOD inhibition rate.

The DH was primarily governed by the linear effects of the three factors (B > C > A) and quadratic effects (A^2^, B^2^), with no significant interaction effects observed. The DPPH scavenging rate and XOD inhibition rates were mainly controlled by the strong quadratic effects of the three factors (A^2^, B^2^, C^2^), while neither linear nor interaction effects were significant. The ADH activation rate was predominantly influenced by significant interaction effects (AB, AC, BC) and quadratic effects (A^2^, B^2^), with no significant linear effects detected. The results were consistent with those of the response surface plot (Figure 3).

Using Design-Expert software for optimization, the optimal enzymatic hydrolysis conditions were determined as follows: an enzyme dosage of 1.35%, hydrolysis time of 2.30 h, and solid-to-liquid ratio of 27.44%. Under these conditions, the DH, DPPH scavenging rates, ADH activation rates and XOD inhibition rates were 48.00%, 55.79%, 12.92%, and 46.61%, respectively.

### 3.3. Bioactivity of Corn Hydrolysates with Different Molecular Weights

**Figure 4:**
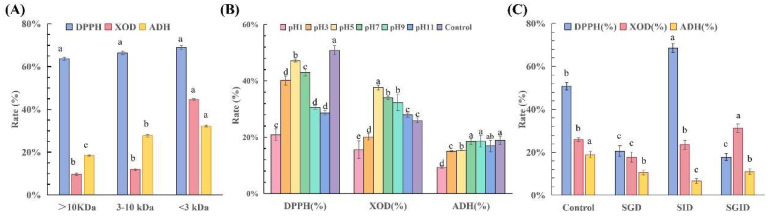
(**A**) Bioactivity of peptide fractions with different molecular weights in corn protein hydrolysates. (**B**) Biological activity of corn peptides with different pH values. (**C**) Biological activity of corn peptides in different simulated digestion treatment groups. Note: Different letters (a–e) indicate significant differences between groups (*p* < 0.05).

The yields of the >10 kDa, 3–10 kDa, and <3 kDa fractions were 41.2%, 23.4%, and 35.4%, respectively. The DPPH radical scavenging rate, ADH activation rate, and XOD inhibition rate of the three fractions (>10 kDa, 3–10 kDa, and <3 kDa) obtained from the ultrafiltration separation of the corn enzymatic hydrolysate were determined (Figure 4A). As the molecular weight of the corn bioactive peptide fractions decreased, their free radical scavenging ability, ADH activation capacity, and XOD inhibition capacity gradually increased. Among them, the <3 kDa fraction exhibited the most excellent bioactivity, with a DPPH radical scavenging rate of 68.88%, an ADH activation rate of 32.23%, and an XOD inhibition rate of 44.64%. This is primarily attributed to the lower steric hindrance and higher flexibility of the lower-molecular-weight corn active peptides [32], which allowed their active groups to be more readily exposed and facilitated their access to the active pockets of ADH/XOD to exert their effects.

### 3.4. Stability of <3 kDa Corn Peptides

#### 3.4.1. Chemical Stability

Following treatment at different pH values (Figure 4B), the overall bioactivity of the <3 kDa corn peptides increased to a peak at pH 5.0 and then declined. The DPPH clearance rate of the <3 kDa corn peptides peaked at pH 5 (47.18%) and reached its lowest point at pH 1 (20.90%). The DPPH clearance rate at pH 5 was closest to that of the control group (50.87%), while strong acidity (pH 1) resulted in an approximately 60% loss of activity, indicating that the antioxidant peptides were highly unstable under strongly acidic conditions. The optimal pH range was between pH 5 and 7, within which 85–93% of the activity was retained. For XOD inhibition activity, the values observed at pH 5–11 were all higher than those of the control group (25.94%), with the maximum inhibition rate (37.59%) occurring at pH 5, indicating that a weakly acidic environment may enhance its inhibitory effect. Regarding ADH activation, all pH-treated groups showed lower activity than the control (18.95%), with the highest activation rate observed at pH 9 (18.59%). Overall, corn peptides were the most stable in neutral to weakly alkaline conditions, while strongly acidic environments caused the most severe damage to their activity.

#### 3.4.2. Biological Stability

As shown in Figure 4C, for DPPH radical scavenging activity, the SID group (68.55%) showed a significantly higher value than the control group (50.87%), while the SGD (20.60%) and SGID (17.76%) groups showed significantly lower values than the control group. This indicates that the SID group may have released more antioxidant peptides or enhanced their activity. The simulated gastric environment likely disrupted the original antioxidant components or generated inhibitory substances. Compared with the control group (25.94% XOD inhibition efficiency), the inhibition rates of the SGD group (17.82%) and SID group (23.51%) slightly decreased, while that of the SGID group (31.15%) slightly increased. Based on these observations, we speculate that the complete gastrointestinal digestion process may have induced conformational changes in the peptides that affect their inhibitory activity or, alternatively, generated new peptide sequences with enhanced XOD inhibitory potential due to the differing cleavage specificities of pepsin and trypsin compared to the alkaline protease used initially.

A similar trend was observed for the activation of ADH. Compared with the control group (18.95%), ADH activation efficiency was reduced in the simulated gastric digestion group (SGD, 10.53%), simulated intestinal digestion group (SID, 6.60%), and simulated gastrointestinal digestion group (SGID, 11.00%). Among the treated groups, corn peptides that underwent complete gastrointestinal digestion exhibited the highest activity. Compared to the pH-treated group, the ADH activity of corn peptides was more significantly affected by protease than by pH.

The antioxidant activity was enhanced and remained stable after intestinal-phase digestion. The XOD inhibitory activity exhibited a unique “digestive enhancement effect” with excellent stability. ADH activation showed weaker resistance to digestion and required special protection.

### 3.5. Purification, Screening and Activity Verification of Corn Peptides

#### 3.5.1. Purification of Corn Peptides

**Figure 5:**
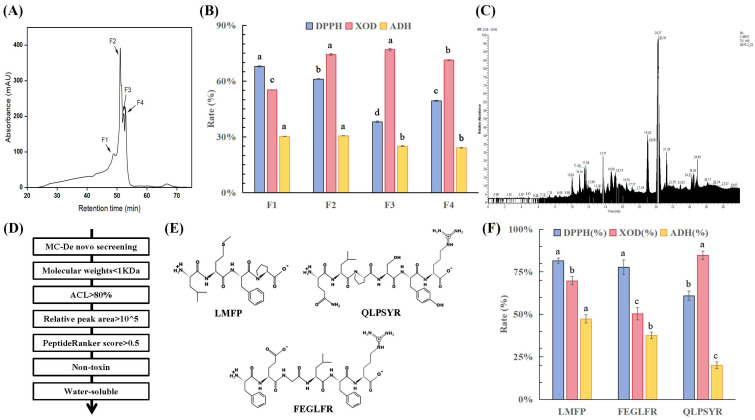
(**A**) Sephadex G-15 gel chromatographic separation diagram. (**B**) Bioactivity of peptide subfractions within <3 kDa component. (**C**) TIC chromatogram. (**D**) Screening flow chart of corn peptides. (**E**) Secondary structure diagrams of screened corn active peptides, and DPPH clearance rate, (**F**) ADH activation rate and XOD inhibition rate of corn bioactive peptides. Note: Different letters (a–d) indicate significant differences between groups (*p* < 0.05).

Four lower-molecular-weight fractions, designated as F1, F2, F3, and F4, were isolated and purified from the <3 kDa corn peptide fraction using gel chromatography (Figure 5A). Activity assays (Figure 5B) revealed that F1 and F2 exhibited strong antioxidant and ADH-activating abilities, with DPPH radical scavenging rates of 67.88% and 61.12% and ADH activation rates of 30.26% and 30.64%, respectively. Meanwhile, F2 and F3 showed stronger UA-lowering activity, with XOD inhibition rates of 74.32% and 76.88%, respectively. Considering all three indicators, F2 displayed outstanding performance across the measured parameters. Therefore, F2 was selected for mass spectrometry identification.

#### 3.5.2. Screening of Corn Peptides

Figure 5C shows the total ion chromatography (TIC) chromatogram of the corn enzymatic hydrolysate F2 fraction. A total of 841 peptide sequences were identified in the F2 fraction. Subsequent screening was performed sequentially based on criteria including molecular weight, ALC, relative peak area, predicted bioactivity score, toxicity score, and solubility (Figure 5D). This process ultimately yielded three corn-derived bioactive peptides: LMFP, FEGLFR, and QLPSYR (Figure 5E). The characteristics of corn peptides, including molecular weight, isoelectric point, charge activity, retention time, relative peak area, activity score, toxicity, and water solubility, are listed in Table 6.

#### 3.5.3. Bioactivity Verification of Screened Corn Active Peptides

The activity profiles of the three key corn peptides (LMFP, FEGLFR, and QLPSYR) are shown in Figure 5F. At a screening concentration of 2.5 mg/mL, all three peptides demonstrated significant antioxidant capacity, with LMFP exhibiting the most outstanding bioactivity, with a DPPH radical scavenging rate of 81.61%, ADH activation rate of 47.35%, and XOD inhibition rate of 69.78%. To further evaluate its potency, a dose–response analysis of LMFP revealed IC_50_ values of 1.6 mg/mL for DPPH radical scavenging and 1.94 mg/mL for XOD inhibition and an EC_50_ value of 2.41 mg/mL for ADH activation.

The amino acid compositions of LMFP, FEGLFR, and QLPSYR are characterized by high levels of aliphatic, aromatic, and basic amino acids, particularly leucine (L), phenylalanine (F), and arginine (R). These amino acids have distinct structural and functional properties. Leucine, with its hydrophobic side chain, can penetrate deeply into the active site of the enzyme. The aromatic ring of phenylalanine not only contributes to radical scavenging but also targets the active pocket of the enzyme, enhancing interaction efficacy. Arginine carries a positive charge, facilitating the formation of hydrogen bonds and strengthening the binding interactions.

### 3.6. Molecular Docking of Simulation of Corn Peptide with XOD and ADH

**Figure 6:**
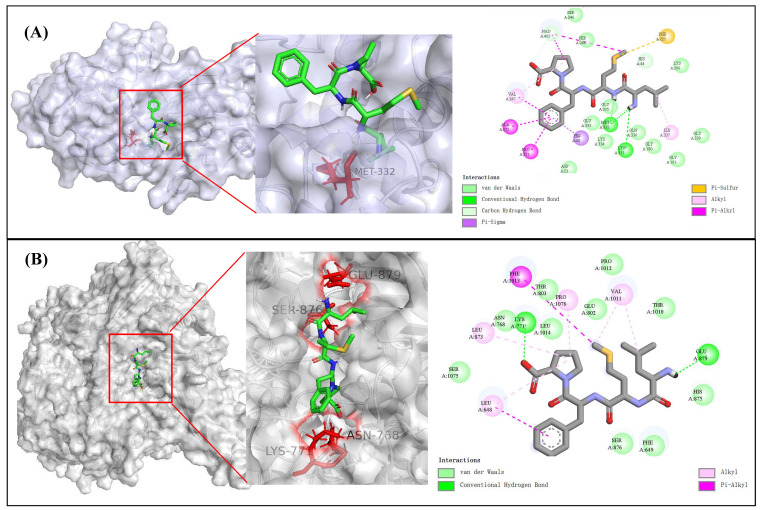
(**A**) The docking sites and interaction forces of corn peptide LMFP with AHD. (**B**) The docking sites and interaction forces of corn peptide LMFP with XOD.

To further understand the molecular recognition and binding mechanisms between the selected corn bioactive peptides and ADH/XOD, molecular docking was employed to simulate the interactions between the receptor and ligands. The active pockets, center coordinates, and binding energies of the corn peptides with the protein crystals 5ENV and 1N5X are listed in Table 7. The binding energies of LMFP, FEGLFR, and QLPSYR to the protein receptors ADH/XOD were all less than −5 kcal/mol, with LMFP exhibiting the lowest value and the strongest binding affinity. The bioactive peptide LMFP formed hydrogen bonds with Lys331 and Met332 in 5ENV. Van der Waals forces were observed with Asp53, Gly180, Gly181, Lys206, Ser246, Ser248, Glu333, Lys334, Gly335, Gln336, Gly339 and NAD+403. Pi–sigma interactions were observed with His48 (Figure 6A). LMFP formed hydrogen bonds with Lys771 and Glu879 in 1N5X, and hydrophobic interactions were identified with Phe649, Asn768, Glu802, Thr803, His875, Ser876, Thr1010, Pro1012, Leu1014, and Ser1075 (Figure 6B). Molecular docking demonstrated that corn peptides primarily bind to ADH and XOD through hydrogen bonds, van der Waals forces, and other interactions. These interactions occur near the active sites of the enzymes, suggesting that the peptides may influence the enzyme activity by occupying or blocking key binding regions.

### 3.7. Limitations and Future Perspectives

The ADH activation assay used in this study is a widely adopted method [25,33,34,35,36]. It measures the initial reaction rates and is suitable for the comparative evaluation of peptide activity. However, peptide-only blanks were not included to correct for potential absorbance interference at 340 nm, and kinetic parameters (e.g., Km and Vmax) were not determined. Consequently, it is difficult to definitively distinguish between true enzymatic activation and possible assay-related artifacts, such as enzyme stabilization. This method is feasible for the preliminary screening of ADH-activating peptides; however, further refinement is required for more in-depth studies. Moreover, the IC_50_/EC_50_ values of LMFP (1.6–2.41 mg/mL) are within the range typical for food-derived peptides but are relatively high compared to pharmaceutical agents. Finally, this study is limited to in vitro and in silico analyses; further in vivo studies are required to validate the physiological relevance. Despite these limitations, our study successfully identified novel corn-derived peptides with multifunctional bioactivities, providing a foundation for future research.

## 4. Conclusions

This study successfully established a process system for preparing corn peptides with integrated alcohol-relieving, UA-reducing, and antioxidant activities. Using the DH, ADH activation rate, XOD inhibition rate, and DPPH radical scavenging rate as indicators, the effects of different proteases and hydrolysis conditions on the activity of corn hydrolysates were investigated. The corn protein hydrolysate prepared with alkaline protease under the conditions of a 1.35% enzyme dosage, 2.30 h hydrolysis time, and 27.44% solid–liquid ratio exhibited the highest activity. Through separation, purification, mass spectrometry identification, and virtual screening, three peptide sequences—LMFP, FEGLFR, and QLPSYR—were obtained, with LMFP demonstrating the strongest activity. This study employed molecular docking technology to elucidate the interaction mechanisms between corn bioactive peptides and ADH/XOD. This study revealed the triple bioactivities of corn germ meal hydrolysate in vitro; however, further work is needed to explore its bioactivities and stability in vivo.

## Figures and Tables

**Table 1:** The results of the response surface experiment.

No.	A: Enzyme Dosage (%)	B: Hydrolysis Times (h)	C: Solid–Liquid Ratios (%)	Y1: DH (%)	Y2: DPPH Radical Scavenging Rate (%)	Y3: ADH Activation Rate (%)	Y4: XOD Inhibition Rate (%)
1	1.25	2.25	27.5	47.28	53.25	11.75	46.25
2	1.25	2.25	27.5	46.34	66.81	12.51	44.7
3	1.5	3	27.5	54.88	12.97	7.74	10.07
4	1.25	2.25	27.5	48.1	64.49	11.27	44.44
5	1.25	2.25	27.5	44.17	59.78	16.13	50.39
6	1.25	3	35	48.99	25.18	9.26	13.67
7	1.25	1.5	20	24.55	29.36	10.14	13.88
8	1.25	2.25	27.5	45.18	62.33	14.78	58.72
9	1.5	1.5	27.5	34.93	13.76	2.18	14.58
10	1	2.25	35	54.21	27.74	10.95	17.52
11	1	2.25	20	40.61	15.95	1.92	3.05
12	1.25	3	20	40.19	22.62	3.05	20.26
13	1.5	2.25	35	56.54	14.74	6.92	22.62
14	1	3	27.5	46.72	17.44	1.89	16.61
15	1.5	2.25	20	44.72	14.69	9.51	18.49
16	1	1.5	27.5	32.55	24.79	7.31	17.58
17	1.25	1.5	35	40.37	22.47	4.28	16.83

**Table 2:** Analysis of variance (ANOVA) table of DH.

Source	Sum of Squares	Df	Mean Square	F-Value	*p*-Value	Significance
Model	1053.40	9	117.04	33.73	<0.0001	**
A	36.04	1	36.04	10.39	0.0146	*
B	426.03	1	426.03	122.77	<0.0001	**
C	313	1	313.00	90.2	<0.0001	**
AB	8.35	1	8.35	2.41	0.1647	
AC	0.79	1	0.79	0.23	0.6474	
BC	12.32	1	12.32	3.55	0.1015	
A^2^	45.17	1	45.17	13.02	0.0086	**
B^2^	219.46	1	219.46	63.24	<0.0001	**
C^2^	0.93	1	0.93	0.27	0.6210	
Residual	24.29	7	3.47			
Lack of fit	14.33	3	4.78	1.92	0.2680	
Pure error	9.96	4	2.49			
Cor total	1077.69	16				
R^2^ = 0.9775 R^2^Adj = 0.9485 C.V. = 4.22%						

Note: * means significant difference (*p* < 0.05); ** means extreme significant difference (*p* < 0.01).

**Table 3:** ANOVA table of DPPH.

Source	Sum of Squares	Df	Mean Square	F-Value	*p*-Value	Significance
Model	6330.12	9	703.35	34.26	<0.0001	**
A	110.71	1	110.71	5.39	0.0532	
B	18.51	1	18.51	0.90	0.3739	
C	7.05	1	7.05	0.34	0.5762	
AB	10.76	1	10.76	0.52	0.4926	
AC	34.46	1	34.46	1.68	0.2362	
BC	22.33	1	22.33	1.09	0.3317	
A^2^	2707.86	1	2707.86	131.91	<0.0001	**
B^2^	1477.46	1	1477.46	71.97	<0.0001	**
C^2^	1317.96	1	1317.96	64.20	<0.0001	**
Residual	143.69	7	20.53			
Lack of fit	34.99	3	11.66	0.43	0.7435	
Pure error	108.70	4	27.18			
Cor total	6473.81	16				
R^2^ = 0.9778 R^2^Adj = 0.9493 C.V. = 14.05%						

Note: ** means extreme significant difference (*p* < 0.01).

**Table 4:** ANOVA table of ADH.

Source	Sum of Squares	Df	Mean Square	F-Value	*p*-Value	Significance
Model	297.07	9	33.01	9.78	0.0033	**
A	2.29	1	2.29	0.68	0.4373	
B	0.49	1	0.49	0.14	0.7158	
C	5.76	1	5.76	1.71	0.2326	
AB	30.14	1	30.14	8.93	0.0203	*
AC	33.76	1	33.76	10.00	0.0159	*
BC	36.42	1	36.42	10.79	0.0134	*
A^2^	65.12	1	65.12	19.30	0.0032	**
B^2^	88.14	1	88.14	26.12	0.0014	**
C^2^	17.36	1	17.36	5.14	0.0577	
Residual	23.62	7	3.37			
Lack of fit	6.28	3	2.09	0.48	0.7122	
Pure error	17.35	4	4.34			
Cor total	320.69	16				
R^2^ = 0.9263 R^2^Adj = 0.8316 C.V. = 22.06%						

Note: * means significant difference (*p* < 0.05); ** means extreme significant difference (*p* < 0.01).

**Table 5:** ANOVA table of XOD.

Source	Sum of Squares	Df	Mean Square	F-Value	*p*-Value	Significance
Model	4054.35	9	450.48	9.63	0.0035	**
A	15.13	1	15.13	0.32	0.5875	
B	0.64	1	0.64	0.01	0.9103	
C	27.98	1	27.98	0.60	0.4647	
AB	3.13	1	3.13	0.07	0.8033	
AC	26.73	1	26.73	0.57	0.4745	
BC	22.75	1	22.75	0.49	0.5081	
A^2^	1284.32	1	1284.32	27.44	0.0012	**
B^2^	1177.79	1	1177.79	25.17	0.0015	**
C^2^	1079.92	1	1079.92	23.08	0.0020	**
Residual	327.60	7	46.80			
Lack of fit	184.39	3	61.46	1.72	0.3008	
Pure error	143.21	4	35.80			
Cor total	4381.95	16				
R^2^ = 0.9253 R^2^Adj = 0.8291 C.V. = 27.07%						

Note: ** means extreme significant difference (*p* < 0.01).

**Table 6:** The characteristics of the corn peptides.

Sequence	LMFP	FEGLFR	QLPSYR
Mass	928	767.87	762.85
Iso-electric point	6.35	6.62	9.55
Net charge	0	0	1
RT	17.3264	13.9416	9.3308
Area	6.40 × 10^5^	1.38 × 10^5^	5.54 × 10^6^
Peptideranker	0.967304	0.8536	0.525549
Toxinpred	Non-Toxin	Non-Toxin	Non-Toxin
Solubility	good	good	good

**Table 7:** Binding energy of molecular docking of corn bioactive peptides with ADH and XOD.

Receptor	Grid Box	Docking Center	Binding Energy (kcal/moL)		
			LMFP	FEGLFR	QLPSYR
ADH (5ENV)	40 Å × 40 Å × 40 Å	−47.3, 44.4, −22.6	−10.44	−6.73	−5.46
XOD (1N5X)	50 Å × 40 Å × 40 Å	96.5, 55.0, 39.5	−7.98	−3.83	−7.91

## Data Availability

The data presented in this study are available in the article. Further inquiries can be directed to the corresponding authors.
